# Diet-Induced Swine Model with Obesity/Leptin Resistance for the Study of Metabolic Syndrome and Type 2 Diabetes

**DOI:** 10.1100/2012/510149

**Published:** 2012-05-02

**Authors:** L. Torres-Rovira, S. Astiz, A. Caro, C. Lopez-Bote, C. Ovilo, P. Pallares, M. L. Perez-Solana, R. Sanchez-Sanchez, A. Gonzalez-Bulnes

**Affiliations:** ^1^Departamento de Reproducción Animal, INIA, 28040 Madrid, Spain; ^2^Departamento de Medicina y Cirugía Animal, Facultad de Veterinaria, UCM, 28040 Madrid, Spain; ^3^Departamento de Producción Animal, Facultad de Veterinaria, UCM, 28040 Madrid, Spain; ^4^Departamento de Mejora Genetica Animal, INIA, 28040 Madrid, Spain

## Abstract

The objective of the present study was to determine the suitability of a swine breed with leptin resistance and predisposition to obesity (the Iberian pig) as model for studies on metabolic syndrome and type 2 diabetes. Thus, six Iberian sows had *ad libitum* access to food enriched with saturated fat (SFAD group; food consumption was estimated to be 4.5 kg/animal/day) whilst four females acted as controls and were fed with 2 kg/animal/day of a commercial maintenance diet. After three months of differential feeding, SFAD animals developed central obesity, dyslipidemia, insulin resistance and impaired glucose tolerance, and elevated blood pressure; the five parameters associated with the metabolic syndrome. Thus, the current study characterizes the Iberian pig as a robust, amenable, and reliable translational model for studies on nutrition-associated diseases.

## 1. Introduction

Obesity is currently declared a global pandemic by the World Health Organization (WHO; http://www.who.int/mediacentre/factsheets/fs311/en/index.html/), with at least 2.6 millions of people dying each year as a result of being overweight or obese. The incidence of overweight and obesity is dramatically rising and, according to WHO predictions, approximately 2.3 billion of people will be overweight and more than 700 million will be obese by the year 2015 (http://www.who.int/features/factfiles/obesity/en/index.html). Furthermore, obesity predisposes to the development of metabolic abnormalities, clustered in the term *metabolic syndrome*. The metabolic syndrome is characterized by the presence of at least three of five symptoms: central obesity, insulin resistance, impaired glucose tolerance, dyslipidemia (increased triglyceridemia and low plasma high-density lipoproteins (HDL) cholesterol), and/or hypertension [1–5]. Moreover, metabolic syndrome is the main risk factor for developing type 2 diabetes [6, 7]. From these considerations, there is an urgent necessity for increasing knowledge about obesity and its effects. However, mechanistical experimentation is not affordable in human beings and animal models are needed.

Most of the experimental studies on obesity and metabolic disorders are carried out on laboratory rodents despite the marked differences in metabolism and adipose tissue biology between rodents and humans [8]. However, different species of large animals offer numerous profitable characteristics [9]. The pig is emerging rapidly as a biomedical model for energy metabolism and obesity in humans because it shares several similarities with humans: omnivorous habits, propensity to sedentary behaviour and fattening, and similar metabolic and cardiovascular features [10–13].

The objective of the present study was to determine the propensity of a swine breed with predisposition to obesity (the Iberian pig) for developing features of metabolic syndrome and type 2 diabetes. The Iberian pig is worldwide known for the production of a unique highly priced dry-cured product, the Iberian ham, with a unique taste due to its abundance in intramuscular fat. In fact, the Iberian pig has a high potential for fat accumulation under its skin and among the muscular fibres [14], due to a polymorphism of the leptin receptor gene (*LEPR*) with effects on food intake, body weight, and fat deposition [15, 16]. As a consequence, Iberian *LEPR* allele increases insatiability and obesity. Such state in human medicine is called *leptin resistance*, the failure, in obese individuals with elevated leptin levels, for suppressing feeding and mediating weight loss [17, 18] due to *LEPR* polymorphisms associated with food preferences and obesity [19]. Thus, having in mind these considerations, our hypothesis was that eating excess and obesity in Iberian pigs would develop a condition similar to the human metabolic syndrome. The final purpose of our study was to characterize a suitable pig model for studies on leptin resistance, obesity, metabolic syndrome, and type 2 diabetes.

## 2. Material and Methods

### 2.1. Animals and Handling

Ten adult Iberian sows (2-3 years old) were used. All the animals had been genotyped for polymorphism on *LEPR* gene with protocols previously described [15] and found to be homozygous for the allele LEPRc.1987T, previously associated with increased appetite, fattening, and bodyweight [15, 16]. The experimental procedure was performed in collective pens at the facilities of the INIA Animal Laboratory Unit (Madrid, Spain). The INIA Animal Unit meets the requirements of the European Union for Scientific Procedure Establishments. The experiment was carried out under Project License from the INIA Scientific Ethic Committee. Animal manipulations were performed according to the Spanish Policy for Animal Protection RD1201/05, which meets the European Union Directive 86/609 about the protection of animals used in experimentation.

Animals were fed, prior to the experimental procedure, with a standard grain-based diet fulfilling their daily maintenance requirements (2 kg/animal/day); mean values of the diet were 89.8% of dry matter, 15.1% of crude protein, and 2.8% of polyunsaturated fat. At the beginning of the experimental procedure, the animals were divided in two different pens corresponding to different diets. Four of the sows acted as controls (control group or group C) and continued being fed with the same diet and amount. The remaining six animals had *ad libitum* access to the same diet but enriched with saturated fat (3.7%; *saturated fat ad libitum* group or group SFAD); during the experimental period (100 days), food consumption was estimated to be 4.5 kg/animal/day.

### 2.2. Evaluation of Body Weight, Size, and Fatness

Body weight, thoracic and abdominal circumferences (obtained with a measuring tape) and back-fat depth (ultrasonically determined at P2 point, at the level of the head of the last right rib) were measured at days 0, 45, and 90 after starting the differential feeding. Thoracic circumference has been shown to be predictive for the amount of carcass fat whilst abdominal circumference is predictive of visceral and subcutaneous fat, obtained by quantitative dissection, in pigs and minipigs [20–22].

### 2.3. Evaluation of Metabolic Status

Blood samples were drawn concurrently with body measures, after a fasting period of around 18 hours, by jugular venopuncture with 5 mL sterile heparin blood vacuum tubes (Vacutainer Systems Europe). Immediately after recovery, the blood was centrifuged at 1500 g for 15 min and the plasma was separated and stored at −20°C until assayed.

Parameters related to metabolism of lipids (triglycerides, total cholesterol, high-density lipoproteins cholesterol (HDL-c) and low-density lipoproteins cholesterol (LDL-c)) were measured with a clinical chemistry analyzer (Screen Point, Hospitex Diagnostics, Sesto Fiorentino, Italy). Plasma HDL-c ratio and LDL-c ratio were calculated by dividing total cholesterol by HDL-c and LDL-c concentrations, respectively; plasma LDL-c/HDL-c ratio was obtained by dividing LDL-c levels by HDL-c concentrations.

Parameters related to metabolism of glucose (glucose and insulin) were measured with a clinical chemistry analyzer for glucose (Screen Point, Hospitex Diagnostics, Sesto Fiorentino, Italia) and with a Porcine Insulin ELISA kit (Mercodia AB, Uppsala, Sweden), respectively. The assay sensitivity was 0.26 UI/L; the intraassay variation coefficient was 3.5%.

Possible changes in *β*-cell function and insulin resistance (IR) during the experimental protocol were assessed by the homeostasis model assessment (HOMA), using the equations HOMA-IR = (FINS × FPG)/22.5 to assess insulin resistance [23] and HOMA-*β* = (20 × FINS)/(FPG − 3.5) to assess beta cell function [24]; FINS is fasting plasma insulin concentration in U/L and FPG is fasting plasma glucose concentration in mmol/L. Furthermore, an oral glucose tolerance test (OGTT) was performed at day 100 after the beginning of experimental procedure, by using the protocol described by Liu et al. [25]. In brief, animals were given 2 g/kg live weight of D-glucose, after a fasting period of around 18 hours, by gavage through a gastric tube inserted into the stomach. Blood samples were obtained for determining plasma glucose and insulin at 0, 15, 30, 60, 90, and 120 minutes after glucose administration, centrifuged, and stored at −20°C until assayed.

### 2.4. Measurement of Cardiovascular Features

Blood pressures were monitored at the beginning and end of the experimental period (days 0 and 100) by using a tail cuff sphygmomanometer.

### 2.5. Statistical Analyses

Effects of diet on body, metabolic and cardiovascular features were assessed by analysis of variance for repeated measures (split-plot ANOVA). Results were expressed as the mean ± SEM, and statistical significance was accepted from *P* < 0.05.

## 3. Results

### 3.1. Effects of Diet on Body Weight and Fatness: Central Obesity

**Figure 1:**
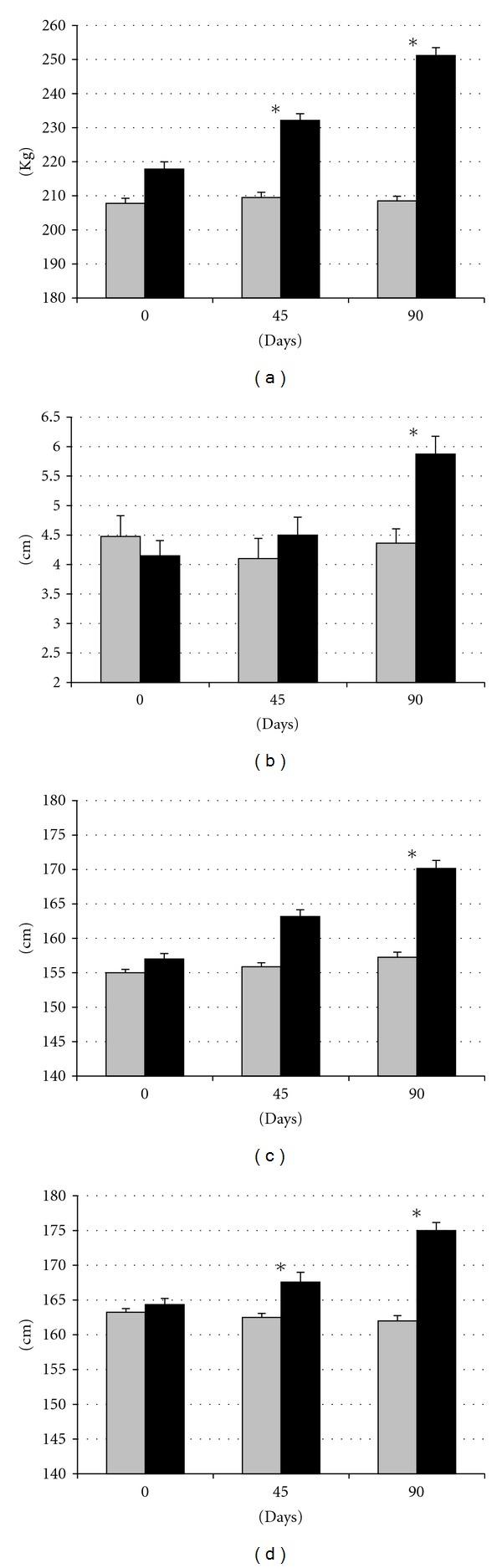
Changes in mean live weight (a), back-fat depth (b), and abdominal and thoracic circumferences ((c) and (d), resp.) over time after differential feeding in control sows (grey bars) and sows with *ad libitum* access to a diet enriched with saturated fat (black bars). Asterisks indicate significant differences.

Live body weight, back-fat depth, and measurement of thoracic and abdominal circumferences were similar between groups at starting the differential feeding period (Figure 1). Values remained unchanged in control animals throughout the experimental period (with increases around 750 g for weight, 3 cm for the circumferences, and 1 mm for back-fat depth) but increased with time in the group SFAD (around 35 kg for weight, 13 and 6 cm for thoracic and abdominal circumferences, resp., and 18 mm for back-fat depth; *P* < 0.05 for body weight and circumferences and *P* < 0.005 for back-fat depth). Differences in body weight and thoracic circumferences between groups reached statistical significance from day 45 onwards (*P* < 0.05), whilst abdominal circumference and back-fat content were different only at day 90 (*P* < 0.05 and *P* < 0.001, resp.). Thus, animals in the SFAD group showed obesity and, specifically, *central obesity* after three months of differential feeding, a first symptom of *metabolic syndrome*.

### 3.2. Effects of Diet on Metabolic Features: Dyslipidemia, Insulin Resistance, and Glucose Intolerance

**Figure 2:**
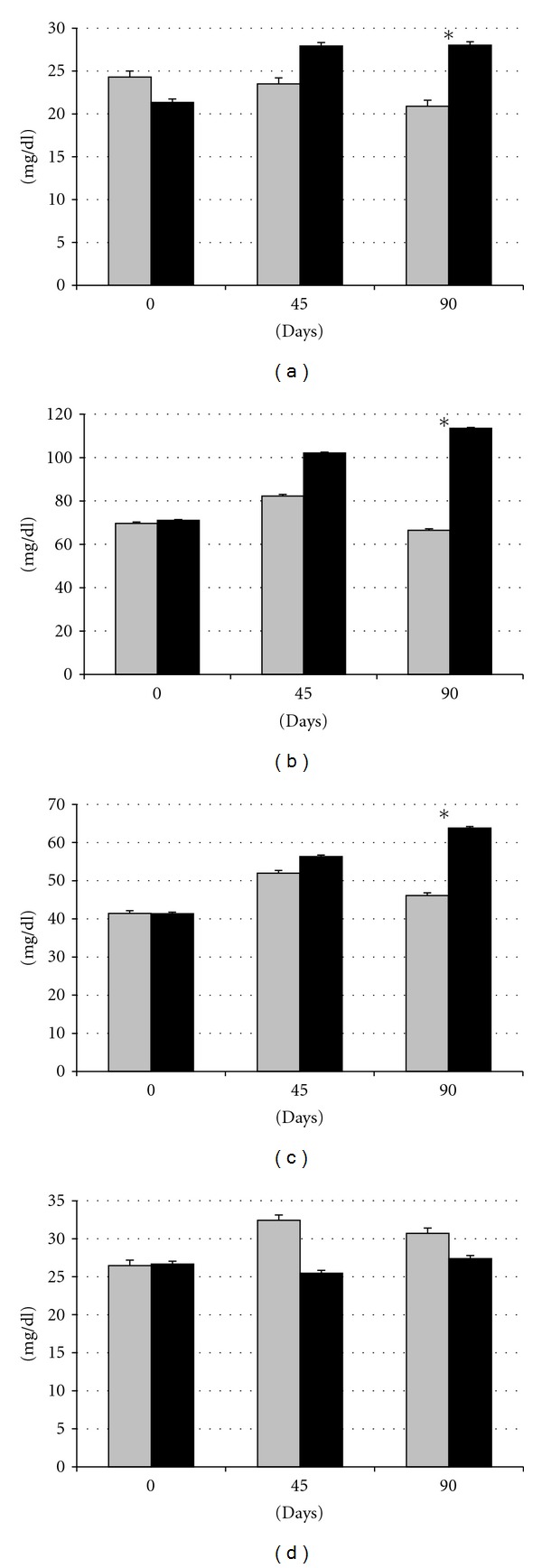
Changes in plasma concentration of triglycerides (a), cholesterol (b), LDL-c (c), and HDL-c (d) over time after differential feeding in control sows (grey bars) and sows with *ad libitum* access to a diet enriched with saturated fat (black bars). Asterisks indicate significant differences.

Assessment of plasma indexes of lipid metabolism showed no significant differences between groups at the beginning of the experimental protocol (Figure 2). After 45 days, differences were not significant. However, after 90 days, SFAD group had significantly (*P* < 0.05) higher plasma levels of triglycerides and cholesterol and higher ratios of HDL-c (total cholesterol divided by HDL-c) and LDL-c/HDL-c (LDL-c levels divided by HDL-c concentrations), since the increase in total cholesterol was accompanied by the increase in LDL-C but not in HDL-c. Such enlarged triglyceridemia with low HDL-c ratios evidenced *dyslipidemia, *which is a second symptom of *metabolic syndrome*.

**Figure 3:**
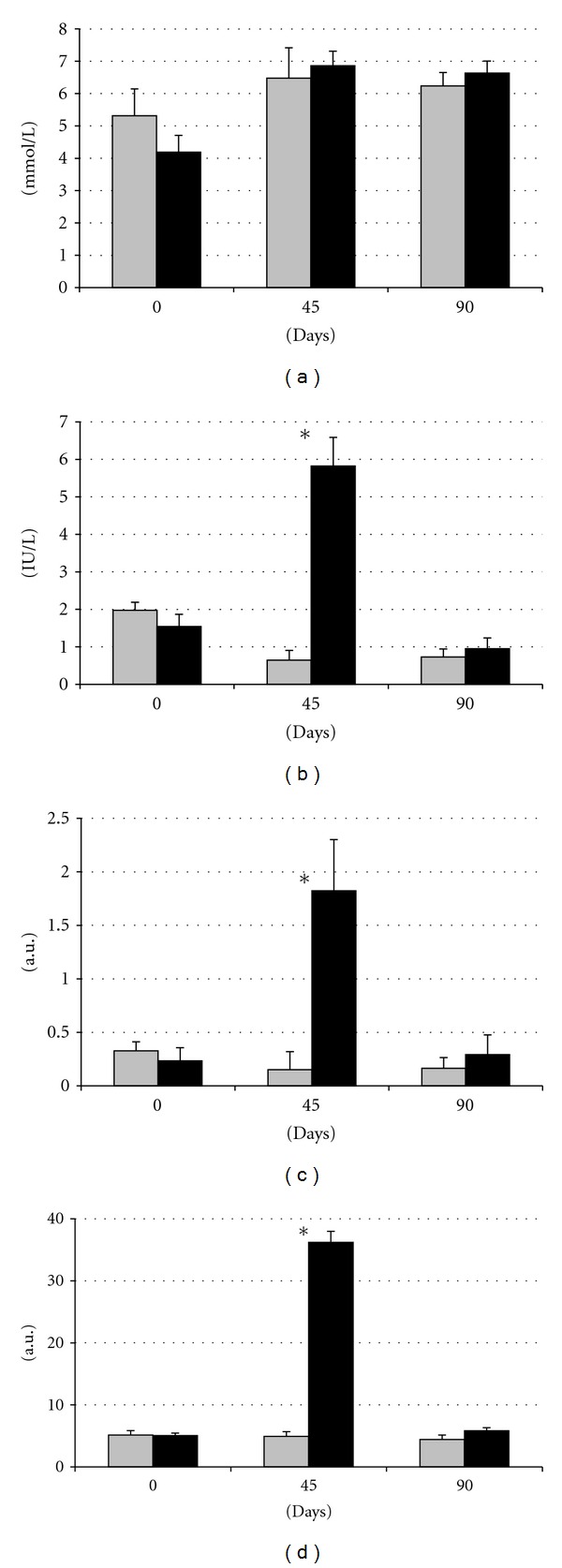
Changes in plasma concentration of glucose (a), insulin (b), and indexes of HOMA-IR and HOMA-*β* ((c) and (d), resp.) over time after differential feeding in control sows (grey bars) and sows with *ad libitum* access to a diet enriched with saturated fat (black bars). Asterisks indicate significant differences.

There were no significant differences between groups in glucose and insulin levels at the beginning of the study (Figure 3). Analysis of plasma glucose showed higher, but not significant, levels in SFAD group after 45 and 90 days of differential feeding. Conversely, plasma insulin concentrations showed different profiles between groups. Insulin level did not significantly differ in group C throughout the experimental period, but plasma insulin concentration increased at day 45 in the group SFAD (*P* < 0.05) and decreased again at day 90 (*P* < 0.01).

Both HOMA-IR and HOMA-*β* indexes remained nearly unchanged throughout the period of study in the group C. Conversely, both indexes increased significantly after 45 days in the group SFAD (*P* < 0.01 for both) and returned to the control values at day 90 (*P* < 0.05 and *P* < 0.01, resp.), when these indexes reached numerically higher but not statistically different values to control females. Thus, both HOMA-IR and HOMA-*β* were higher in SFAD at day 45 (*P* < 0.05 and *P* < 0.01, resp.), evidencing *insulin resistance*, the third symptom of *metabolic syndrome*, and *impaired *β*-cell function*, respectively.

**Figure 4:**
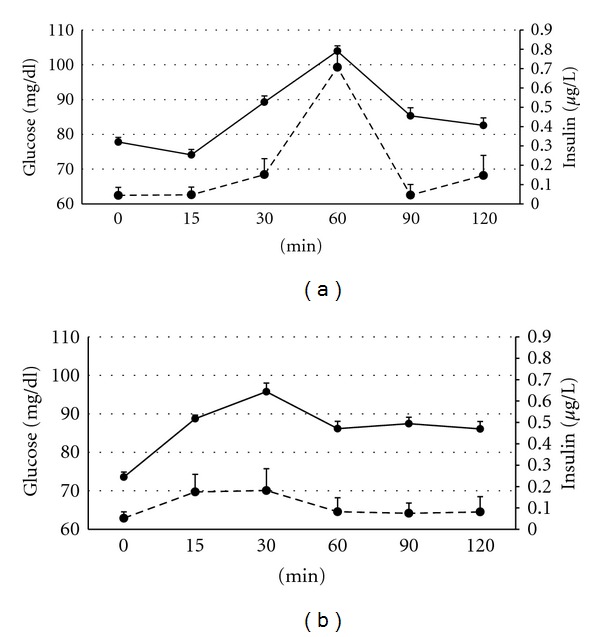
Changes in plasma concentration of glucose (continuous line) and insulin (discontinuous line) over time after oral administration of 2 g/kg live weight of D-glucose in control sows (a) and sows with *ad libitum* access to a diet enriched with saturated fat (b).

The oral glucose tolerance test (OGTT; Figure 4) showed that, in control animals, the plasma glucose levels started to increase after 15 minutes and reached a peak around one hour later (*P* < 0.01) for rapidly declining to starting values afterwards. Assessment of insulin levels in these pigs evidenced a well-characterized acute insulin response. In contrast, the OGTT showed a deficient glucose elimination (i.e., *glucose intolerance*, fourth symptom of the *metabolic syndrome*) in the group SFAD, since glucose levels did not decrease for the period of study. Moreover, SFAD sows had a deficient insulin secretion, confirming the diet-induced *β*-cell dysfunction suggested by prior changes in HOMA-*β* and evidencing the prodrome of *type 2 diabetes*.

### 3.3. Effects of Diet on Cardiovascular Features: Hypertension

**Figure 5:**
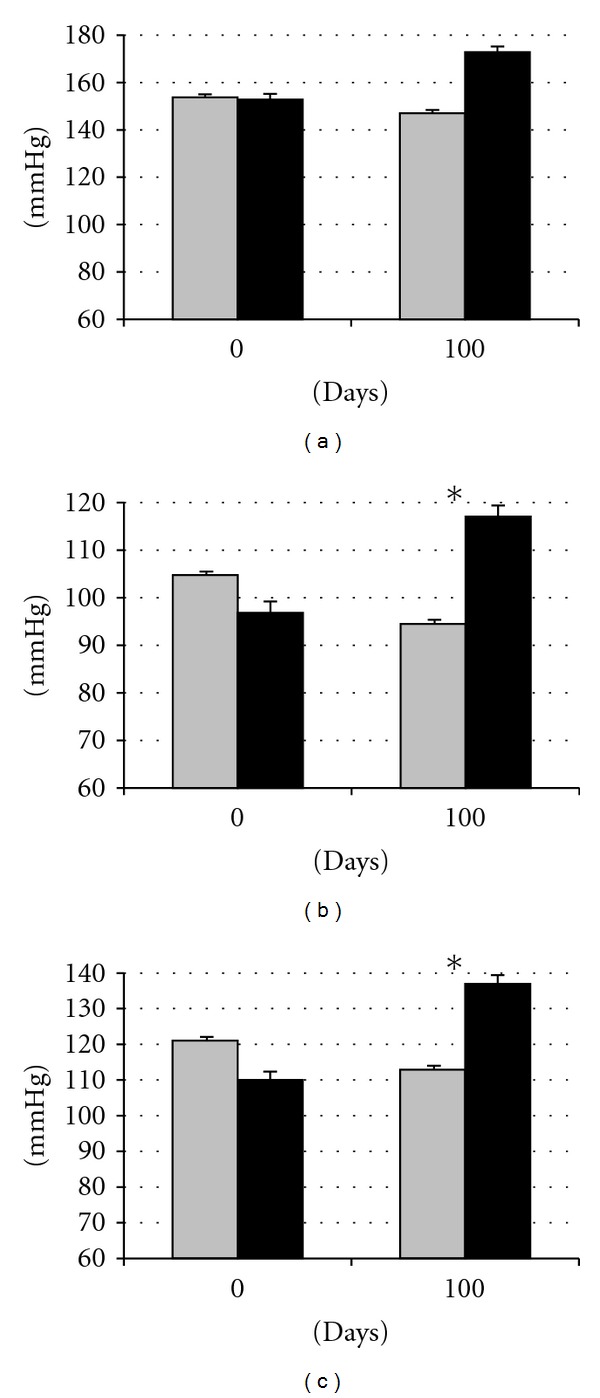
Changes in systolic (a), diastolic (b), and mean (c) blood pressures over time after differential feeding in control sows (grey bars) and sows with *ad libitum* access to a diet enriched with saturated fat (black bars). Asterisks indicate significant differences.

Assessment of blood pressure showed similar values in both groups at the beginning of study (Figure 5). At the end of the treatment, all the parameters remained stable in control females but increased in obese females; these differences were statistically significant for diastolic and mean blood pressure (*P* < 0.01 and *P* < 0.005, resp.) evidencing a *hypertensive state*, the fifth symptom of a *metabolic syndrome*.

## 4. Discussion

The results found in the present study indicate that adult females of the Iberian pig, a swine breed with *leptin resistance*, can develop the prodrome of *metabolic syndrome* and *type 2 diabetes* when allowed to freely eat a diet enriched with saturated fat. This feature was found as early as in three months. Thus, current experiment reinforces previous evidences about the susceptibility of this breed to changes in nutritional inputs [26, 27] and characterizes the Iberian pig as a robust, amenable, and reliable translational model for studies on nutrition-associated diseases.

In these three months, pigs developed alterations in the five parameters associated with the metabolic syndrome. Obesity was associated with different degrees of dyslipidemia, insulin resistance and impaired glucose tolerance, and elevated blood pressure. Such finding reinforces the strength of our model. There are no other animal models consistently showing four or more risk factors of the syndrome [13]. Establishment of the five parameters of the metabolic syndrome has been previously found in the Ossabaw pig [21]. However, it is known that Ossabaw pigs are derived from the Iberian pigs introduced by Spanish and Portuguese colonizers, since there were no pigs in the New World before their arrival in the 15th century. Iberian pigs, conversely to Ossabaw pigs, are numerous (the estimated census of breeding sows, only in Spain, is around 238.000) and, hence, easy to get and reasonably priced. Finally, the robustness of the model is also reinforced by the fact that homeostatic changes were found when feeding the sows with diets having a considerably lower level of fat content and energy than provided in previous studies with other swine breeds [21, 28, 29].

The effects of the diet on body weight, size, and fat content were remarkable and were observed very early after starting the differentiated feeding, but the pattern of fattening seemed to be affected by time. Differences in body measurements were firstly found in body weight and thoracic circumference and thereafter in subcutaneous fat and abdominal circumference. Thus, we can assume that fat deposition when there is a positive energy balance is firstly intramuscular in the whole carcass. At later stages, fat deposition is also subcutaneous and, which is more important, visceral. Visceral deposition of fat gives way to *central obesity*, the former and causal factor for metabolic syndrome.

The current study also indicates that effects on carbohydrate metabolism were found earlier than the effects on lipid metabolism. There were no significant differences in plasma glucose concentrations throughout the study as it has been described in the first stages of the human disease [30]. However, high intake of saturated fat impaired glucose regulation and induced some degree of *insulin resistance* and *altered *β*-cell function* (main parameters of metabolic syndrome and type 2 diabetes) at 45 days after starting the differentiated feeding. This finding is in agreement with previous studies in rats also fed with saturated fat, which developed insulin resistance in a very short term [31–33], but also with observational data in human beings [34]. Animals of the current experiment were capable of counterbalancing thereafter the prodrome of metabolic syndrome and type 2 diabetes as evidenced by the plasma insulin concentrations and HOMA-IR and HOMA-**β** found at day 90. However, the equilibrium was unstable and the challenge of glucose during the OGTT evidenced the weakness of this adaptation; animals confronted with such acute high supply of glucose showed *glucose intolerance*, other parameter of the *metabolic syndrome*, and alterations in *β*-cell function, which are the main cause of *type 2 diabetes* [35].

Establishment of insulin resistance has been linked to elevated levels of triglycerides in blood and tissues [32, 36–38], and *hypertriglyceridemia* is another parameter of metabolic syndrome. However, in the current study, hypertriglyceridemia was found in a later stage than insulin resistance; simultaneously with the increase in visceral fat, which confirms earlier hypothesis indicating that hypertriglyceridemia is related primarily to the amount of such kind of fat [39]. Obese sows of this study showed a well-established dyslipidemic profile with significantly higher plasma triglycerides but also total cholesterol and LDL-c/HDL-c ratio, suggesting higher levels of LDL-c than HDL-c. These features resemble hyperlipidemia type 2 (increased total cholesterol and LDL-c) and the so-called *lipid triad* in human medicine (elevated triglycerides, elevated LDL-c, and low HDL-c), [40, 41]. The rise of cholesterol caused by elevations of LDL-c may occur both in type 1 and type 2 diabetic patients; however, concurrently low HDL-c levels are indicative of obese individuals with type 2 diabetes [42].

Alterations of carbohydrate and lipoprotein metabolism were also early identified as risk factors for *hypertension*, the fifth symptom in the metabolic syndrome [43, 44]. In our study, higher blood pressures were also found in the obese sows, indicating that they have a hyperdynamic circulatory state, similar to previously described in Ossabaw pigs [45]. In our study, changes were mainly found in diastolic pressure, coincidentally with studies in human beings [46, 47]. Hyperdynamic circulatory states, characterized by increased blood pressure, total blood volume, cardiac output, and tissue metabolic demand, have been hypothesized to be the main cause of cardiac and hemodynamic alterations in obese individuals [48–50].

In conclusion, this study indicates that Iberian pigs freely eating saturated fat diets are prone to central obesity, abnormalities in insulin-glucose regulation, dyslipidemia, and elevated blood pressure, the symptoms of metabolic syndrome. Thus, the current data characterize the Iberian pig as a robust, amenable, and reliable translational model for studies on nutrition-associated diseases.

## References

[1] Reaven GM (1988). Role of insulin resistance in human disease. *Diabetes*.

[2] Grundy SM, Brewer HB, Cleeman JI, Smith SC, Lenfant C (2004). Definition of metabolic syndrome: report of the National Heart, Lung, and Blood Institute/American Heart Association conference on scientific issues related to definition. *Circulation*.

[3] Kahn R, Buse J, Ferrannini E, Stern M (2005). The metabolic syndrome: time for a critical appraisal - Joint statement from the American Diabetes Association and the European Association for the Study of Diabetes. *Diabetes Care*.

[4] Olufadi R, Byrne CD (2008). Clinical and laboratory diagnosis of the metabolic syndrome. *Journal of Clinical Pathology*.

[5] Rocha E (2008). Metabolic syndrome: an entity that lacks a clear definition but which is useful to identify in clinical practice. *Portuguese Journal of Cardiology*.

[6] Church TS, Thompson AM, Katzmarzyk PT (2009). Metabolic syndrome and diabetes, alone and in combination, as predictors of cardiovascular disease mortality among men. *Diabetes Care*.

[7] Aschner P (2010). Metabolic syndrome as a risk factor for diabetes. *Expert Review of Cardiovascular Therapy*.

[8] Arner P (2005). Resistin: yet another adipokine tells us that men are not mice. *Diabetologia*.

[9] Lunney JK (2007). Advances in swine biomedical model genomics. *International Journal of Biological Sciences*.

[10] Douglas WR (1972). Of pigs and men and research: a review of applications and analogies of the pig, sus scrofa, in human medical research. *Space Life Sciences*.

[11] Mahley RW, Weisgraber KH, Innerarity T, Brewer HB, Assmann G (1975). Swine lipoproteins and atherosclerosis. Changes in the plasma lipoproteins and apoproteins induced by cholesterol feeding. *Biochemistry*.

[12] Bell FP, Gerrity RG (1992). Evidence for an altered lipid metabolic state in circulating blood monocytes under conditions of hyperlipemia in swine and its implications in arterial lipid metabolism. *Arteriosclerosis and Thrombosis*.

[13] Spurlock ME, Gabler NK (2008). The development of porcine models of obesity and the metabolic syndrome. *Journal of Nutrition*.

[14] Nieto R, Miranda A, García MA, Aguilera JF (2002). The effect of dietary protein content and feeding level on the rate of protein deposition and energy utilization in growing Iberian pigs from 15 to 50 kg body weight. *British Journal of Nutrition*.

[15] Óvilo C, Fernández A, Noguera JL (2005). Fine mapping of porcine chromosome 6 QTL and LEPR effects on body composition in multiple generations of an Iberian by Landrace intercross. *Genetical Research*.

[16] Muñoz G, Ovilo C, Silló L, Tomás A, Noguera JL, Rodríguez MC (2009). Single- And joint-population analyses of two experimental pig crosses to confirm quantitative trait loci on Sus scrofa chromosome 6 and leptin receptor effects on fatness and growth traits. *Journal of Animal Science*.

[17] Martin SS, Qasim A, Reilly MP (2008). Leptin resistance. A possible interface of inflammation and metabolism in obesity-related cardiovascular disease. *Journal of the American College of Cardiology*.

[18] Myers MG, Cowley MA, Münzberg H (2008). Mechanisms of leptin action and leptin resistance. *Annual Review of Physiology*.

[19] Mizuta E, Kokubo Y, Yamanaka I (2008). Leptin gene and Leptin receptor gene polymorphisms are associated with sweet preference and Obesity. *Hypertension Research*.

[20] Witczak CA, Mokelke EA, Boullion R, Wenzel J, Keisler DH, Sturek M (2005). Noninvasive measures of body fat percentage in male Yucatan swine. *Comparative Medicine*.

[21] Dyson MC, Alloosh M, Vuchetich JP, Mokelke EA, Sturek M (2006). Components of metabolic syndrome and coronary artery disease in female Ossabaw swine fed excess atherogenic diet. *Comparative Medicine*.

[22] Christoffersen BO, Grand N, Golozoubova V, Svendsen O, Raun K (2007). Gender-associated differences in metabolic syndrome-related parameters in Göttingen Minipigs. *Comparative Medicine*.

[23] Matthews D, Hosker J, Rudenski A, Naylor B, Treacher D, Turner R (1985). Homeostasis model assessment: insulin resistance and B-cell function from fasting plasma glucose and insulin concentrations in man. *Diabetologia*.

[24] Wallace TM, Levy JC, Matthews DR (2004). Use and abuse of HOMA modeling. *Diabetes Care*.

[25] Liu Y, Wang Z, Yin W (2007). Severe insulin resistance and moderate glomerulosclerosis in a minipig model induced by high-fat/high-sucrose/high-cholesterol diet. *Experimental Animals*.

[26] Torres-Rovira L, Pallares P, Vigo E (2011). Plasma leptin, ghrelin and indexes of glucose and lipid metabolism in relation to the appearance of post-weaning oestrus in mediterranean obese sows (Iberian pig). *Reproduction in Domestic Animals*.

[27] Torres-Rovira L, Pallares P, Gonzalez-Añover P, Perez-Solana ML, Gonzalez-Bulnes A (2011). The effects of age and reproductive status on blood parameters of carbohydrate and lipid metabolism in Iberian obese sows. *Reproductive Biology*.

[28] Dixon JL, Stoops JD, Parker JL, Laughlin MH, Weisman GA, Sturek M (1999). Dyslipidemia and vascular dysfunction in diabetic pigs fed an atherogenic diet. *Arteriosclerosis, Thrombosis, and Vascular Biology*.

[29] Boullion RD, Mokelke EA, Wamhoff BR (2003). Porcine model of diabetic dyslipidemia: insulin and feed algorithms for mimicking diabetes mellitus in humans. *Comparative Medicine*.

[30] Kahn SE, Prigeon RL, McCulloch DK (1993). Quantification of the relationship between insulin sensitivity and *β*- cell function in human subjects: evidence for a hyperbolic function. *Diabetes*.

[31] Kraegen EW, Clark PW, Jenkins AB, Daley EA, Chisholm DJ, Storlien LH (1991). Development of muscle insulin resistance after liver insulin resistance in high-fat-fed rats. *Diabetes*.

[32] Storlien LH, Jenkins AB, Chisholm DJ, Pascoe WS, Khouri S, Kraegen EW (1991). Influence of dietary fat composition on development of insulin resistance in rats. Relationship to muscle triglyceride and *ω*-3 fatty acids in muscle phospholipid. *Diabetes*.

[33] Dimopoulos N, Watson M, Sakamoto K, Hundal HS (2006). Differential effects of palmitate and palmitoleate on insulin action and glucose utilization in rat L6 skeletal muscle cells. *Biochemical Journal*.

[34] Morrison CD, Huypens P, Stewart LK, Gettys TW (2009). Implications of crosstalk between leptin and insulin signaling during the development of diet-induced obesity. *Biochimica et Biophysica Acta*.

[35] Prentki M, Nolan CJ (2006). Islet *β* cell failure in type 2 diabetes. *Journal of Clinical Investigation*.

[36] Roden M, Price TB, Perseghin G (1996). Mechanism of free fatty acid-induced insulin resistance in humans. *Journal of Clinical Investigation*.

[37] Koyama K, Chen G, Lee Y, Unger RH (1997). Tissue triglycerides, insulin resistance, and insulin production: implications for hyperinsulinemia of obesity. *American Journal of Physiology*.

[38] Schinner S, Scherbaum WA, Bornstein SR, Barthel A (2005). Molecular mechanisms of insulin resistance. *Diabetic Medicine*.

[39] Despres JP (1994). Dyslipidaemia and obesity. *Bailliere’s Clinical Endocrinology and Metabolism*.

[40] Temelkova-Kurktschiev T, Hanefeld M (2004). The lipid triad in type 2 diabetes—prevalence and relevance of hypertriglyceridaemia/low high-density lipoprotein syndrome in type 2 diabetes. *Experimental and Clinical Endocrinology and Diabetes*.

[41] Nesto RW (2005). Beyond low-density lipoprotein: addressing the atherogenic lipid triad in type 2 diabetes mellitus and the metabolic syndrome. *American Journal of Cardiovascular Drugs*.

[42] Goldberg RB (1981). Lipid disorders in diabetes. *Diabetes Care*.

[43] Krzesinski JM, Carlier PG, Rorive GL (1988). Interrelationship of hypertension, plasma lipids and atherosclerosis. *Drugs*.

[44] Reaven GM (1990). Role of abnormalities of carbohydrate and lipoprotein metabolism in the pathogenesis and clinical course of hypertension. *Journal of Cardiovascular Pharmacology*.

[45] Dincer UD (2011 ). Cardiac *β*-adrenoceptor expression is markedly depressed in Ossabaw swine model of cardiometabolic risk. *International Journal of General Medicine*.

[46] Mulé G, Cottone S, Nardi E, Andronico G, Cerasola G (2006). Metabolic syndrome in subjects with essential hypertension: relationships with subclinical cardiovascular and renal damage. *Minerva Cardioangiologica*.

[47] Klaus JR, Hurwitz BE, Llabre MM (2009). Central obesity and insulin resistance in the cardiometabolic syndrome: pathways to preclinical cardiovascular structure and function. *Journal of the CardioMetabolic Syndrome*.

[48] Carroll JF, Jones AE, Hester RL, Reinhart GA, Cockrell K, Mizelle HL (1997). Reduced cardiac contractile responsiveness to isoproterenol in obese rabbits. *Hypertension*.

[49] Hall JE, Brands MW, Zappe DH (1995). Hemodynamic and renal responses to chronic hyperinsulinemia in obese, insulin-resistant dogs. *Hypertension*.

[50] Hall JE, Brands MW, Zappe DH, Galicia MA (1995). Insulin resistance, hyperinsulinemia, and hypertension: causes, consequences, or merely correlations?. *Proceedings of the Society for Experimental Biology and Medicine*.

